# The Influence of Lactoferrin in Plasma and Peritoneal Fluid on Iron Metabolism in Women with Endometriosis

**DOI:** 10.3390/ijms24021619

**Published:** 2023-01-13

**Authors:** Ewa Skarżyńska, Monika Wróbel, Hanna Zborowska, Mateusz Franciszek Kołek, Grzegorz Mańka, Mariusz Kiecka, Michał Lipa, Damian Warzecha, Robert Spaczyński, Piotr Piekarski, Beata Banaszewska, Artur Jakimiuk, Tadeusz Issat, Wojciech Rokita, Jakub Młodawski, Maria Szubert, Piotr Sieroszewski, Grzegorz Raba, Kamil Szczupak, Tomasz Kluz, Marek Kluza, Mirosław Wielgoś, Barbara Lisowska-Myjak, Piotr Laudański

**Affiliations:** 1 Department of Biochemistry and Clinical Chemistry, Medical University of Warsaw, 02-097 Warsaw, Poland; 2 1st Department of Obstetrics and Gynecology, Medical University of Warsaw, 02-015 Warsaw, Poland; 3 Department of Laboratory Medicine, Medical University of Warsaw, 02-097 Warsaw, Poland; 4 Faculty of Medicine, Medical University of Vienna, 1090 Vienna, Austria; 5 Angelius Provita Hospital, 40-611 Katowice, Poland; 6 Center for Gynecology, Obstetrics and Infertility Treatment Pastelova, 60-198 Poznan, Poland; 7 Division of Infertility and Reproductive Endocrinology, Department of Gynecology, Obstetrics and Gynecological Oncology, Poznan University of Medical Sciences, 61-701 Poznan, Poland; 8 Chair and Department of Laboratory Diagnostics, Poznan University of Medical Sciences, 61-701 Poznan, Poland; 9 Department of Reproductive Health, Institute of Mother and Child in Warsaw, 01-211 Warsaw, Poland; 10 Department of Obstetrics and Gynecology, Central Clinical Hospital of the Ministry of Interior, 02-507 Warsaw, Poland; 11 Department of Obstetrics and Gynecology, Institute of Mother and Child in Warsaw, 01-211 Warsaw, Poland; 12 Collegium Medicum, Jan Kochanowski University in Kielce, 02-369 Kielce, Poland; 13 Clinic of Obstetrics and Gynecology, Provincial Combined Hospital in Kielce, 25-736 Kielce, Poland; 14 Department of Gynecology, Obstetrics Medical University of Lodz, 90-419 Lodz, Poland; 15 Department of Surgical Gynecology and Oncology, Medical University of Lodz, 90-419 Lodz, Poland; 16 Department of Fetal Medicine and Gynecology, Medical University of Lodz, 90-419 Lodz, Poland; 17 Clinic of Obstetric and Gynecology in Przemysl, 37-700 Przemysl, Poland; 18 Department of Obstetrics and Gynecology, University of Rzeszow, 35-310 Rzeszow, Poland; 19 Department of Gynecology, Gynecology Oncology and Obstetrics, Institute of Medical Sciences, Medical College of Rzeszow University, 35-310 Rzeszow, Poland; 20 Department of Obstetrics, Gynecology and Gynecological Oncology, Medical University of Warsaw, 02-091 Warsaw, Poland; 21 Women’s Health Research Institute, Calisia University, 62-800 Kalisz, Poland; 22 OVIklinika Infertility Center, 01-377 Warsaw, Poland

**Keywords:** ferritin, iron, lactoferrin, transferrin, endometriosis

## Abstract

The aim of this study was to investigate the relationship between lactoferrin and iron and its binding proteins in women with endometriosis by simultaneously measuring these parameters in plasma and peritoneal fluid. Ninety women were evaluated, of whom 57 were confirmed as having endometriosis. Lactoferrin was measured by ELISA, transferrin, ferritin and iron on a Cobas 8000 analyser. Lactoferrin and transferrin in peritoneal fluid were lower compared to plasma, in contrast to ferritin and iron. In plasma, lactoferrin showeds associations with iron and transferrin in endometriosis and with ferritin in the group without endometriosis. Lactoferrin in peritoneal fluid correlated with lactoferrin, iron and transferrin of plasma in patients without endometriosis. The ratio of lactoferrin concentration in peritoneal fluid to plasma differentiated stage I versus IV of endometriosis and was negatively correlated with the iron ratio in patients without endometriosis. The ferritin ratio differentiated women with and without endometriosis. The very high ferritin ratios, especially in advanced stages of endometriosis, suggest the protective involvement of this protein in peritoneal fluid and the loss of this role by lactoferrin. The results demonstrate the validity of assessing iron metabolism in women with endometriosis, which may be useful as a marker of the disease and its progression.

## 1. Introduction

Endometriosis is caused by the presence of endometrial-type tissue outside the uterus and affects 1 in 10 individuals of reproductive age worldwide [1]. It manifests variously, depending on the location, as pelvic pain, urinary and gastrointestinal symptoms, in some cases its presence can be one of the causes of infertility [2]. According to the most popular theory proposed by Sampson, endometriosis is a result of retrograde menstruation [3].

The diagnosis of endometriosis remains a challenging problem and leads to delays of 5 to more than 7 years [4,5,6]. The high prevalence of this disease in the population of patients of reproductive age [7] and the long asymptomatic period preceding the onset of clinical symptoms indicate the need to search for diagnostic parameters to facilitate targeted therapeutic decisions [6]. Current laboratory practice does not provide specific markers for detecting and assessing the advancement of endometriosis in either plasma or peritoneal fluid. The consideration of peritoneal fluid is based on reports confirming its diagnostic potential, for example, in assessing the prognosis of gastric- cancer patients [8]. An interesting report was recently presented by Atta et al. showing that in patients with cirrhosis, peritoneal fluid is a better prognostic tool than serum in assessing the possibility of transformation to hepatocellular carcinoma [9] Therefore, peritoneal fluid is a valuable material that can, despite the need for invasive collection, have clinical significance.

The pathogenesis of endometriosis has been investigated from a molecular perspective, including the involvement of many biologically active molecules such as proteases, chemokines and miRNAs assessed in plasma and peritoneal fluid, in which they may show differential expression [10,11,12,13,14].

Iron (Fe) homeostasis, including the involvement of specific proteins [15,16,17,18,19,20,21], is directly related to the pathophysiology of endometriosis. In the course of endometriosis there is a correlation between the release of iron from erythrocytes, apoptotic endometrial tissue and cell debris which sweep into the peritoneal cavity and create a pro-inflammatory and pro-oxidative environment [22,23,24,25].

Iron is an essential metal for functioning of living organisms and is linked to numerous enzymes and proteins. Its excessive accumulation in cells, tissues and organs is toxic and leads to various diseases [22]. Iron overload caused by retrograde menstruation and periodic hemorrhage from ectopic lesions in the peritoneal fluid is an important factor promoting the inflammatory microenvironment [22,23].

The maintenance of iron concentration in physiological equilibrium depends, among various factors, on the interaction of the diverse properties of iron-binding proteins including lactoferrin (LF), transferrin (TF) and ferritin (FT). Lactoferrin is considered as a critical control point in physiological homeostasis [26].

Lactoferrin (LF, lactotransferrin) is a nonheme multifunctional cationic natural protein found in exocrine secretions and in the secondary granules of neutrophils [26,27,28,29]. The characteristic functions of this protein includes binding of toxic free-iron. Compared to transferrin, only LF shows the ability to sequester iron even at low pH (around 3.0) which is common in infected and inflamed tissues [20,27,29,30]. Another important function of LF is reduction of oxidative stress. It has been pointed out [16,28] that its antioxidant activity may be complementary to FT and TF and together form a coordinated system of iron-regulating proteins. This protein also prevents the growth of pathogens that require iron for survival and replication. LF is a key component of host defense and modulates innate and adaptive immunity [26,31,32].

LF does not deliver iron to cells,-in contrast to transferrin, the transport function of LF is not known [20]. This protein is physiologically localized inside secondary granules of neutrophils [27,29] and, like ferritin [15,21], is a physiological intracellular protein with possible expression in response to oxidative stress and the ability to be secreted into the extracellular matrix upon neutrophil activation. LF is involved in the regulation of inflammatory processes, and shows an increase in concentration parallel to FT (positive acute phase protein) and opposite to TF (negative acute phase protein) [33].

Transferrin (TF) is a transport protein with a similar sequence and structure to LF. It removes toxic free-iron from the blood and delivers it to cells [18,20].

Ferritin (FT), as an iron-storage protein, is beneficial for its metabolism because it sequesters iron during its excess limiting its pro-oxidative activity. Under certain conditions, it can also release iron with pro-oxidative activity, which may be related to the unregulated release of iron from ferritin [15,21].

In general, LF, TF and FT protect the host against the reactive oxygen species (ROS) induced by an excess of free iron. The aim of this study was to investigate whether, and within what range, LF works in conjunction with iron parameters (Fe, TF, FT) in women with endometriosis. Simultaneous determination in plasma and peritoneal fluid was used to compare these specific environments. Calculated values of peritoneal fluid to plasma concentration ratios were used to determine the relationships observed in the progression of endometriosis.

## 2. Results

Table 1 presents the characteristics of the study and control group in terms of age, presenting symptoms, stage of endometriosis and cycle phase. Due to the clinical complexity of endometriosis, the numbers of patients in each group concerning the presence of specific symptoms was not equal to those in the control and study groups.

**Figure 1:**
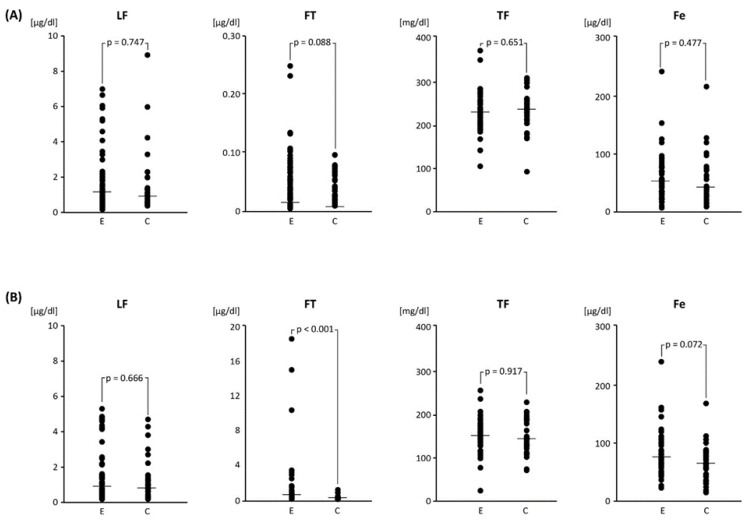
Comparison of concentrations of tested variables:; lactoferrin (LF), ferritin (FT), transferrin (TF) and iron (Fe) between the group of with (E, *n* = 57) and without (C, *n* = 33) endometriosis, respectively, for plasma (**A**) and peritoneal fluid (**B**). *p*-values from *U*-MannWhitney test for independent observations between group with endometriosis (E) and without endometriosis (C) for plasma (**A**) and peritoneal fluid (**B**). The dots represent a single score, the dash represents the median.

Table 2 compares the concentrations of LF, FT, TF and Fe determined in plasma and peritoneal fluid in the group of women enrolled in the study and in the groups of women with and without endometriosis that were distinguished from them. Transferrin concentrations were significantly higher in plasma compared to peritoneal fluid in contrast to ferritin and Fe that were significantly higher in peritoneal fluid, *p* < 0.05 (only Fe in the group without endometriosis higher, but without statistical significance). Significant differences in concentrations between the group with endometriosis (E) and without endometriosis (C) were found only in peritoneal fluid for ferritin (Figure 1).

Table 3 compares the concentrations of measured iron metabolism parameters determined in plasma and peritoneal fluid in four subsequent stages of endometriosis. The studied iron metabolism parameters measured in plasma dido not differentiate the degree of endometriosis (*p* > 0.05). In contrast, in the peritoneal fluid, ferritin and Fe concentrations showed an increase in subsequent stages of the disease.

Table 4 and Table 5 presents the statistically significant correlation coefficients (Rs) of Spearman’s rank order for the studied parameters in plasma and peritoneal fluid in the group without and with endometriosis (Table 4) and in the group with mild (stages I and II) and severe (stages III and IV) endometriosis (Table 5).

Significant correlations of LF with Fe and with TF in the group with endometriosis and LF with FT in the group without endometriosis were obtained in plasma with no significant correlations of LF with these parameters in peritoneal fluid. LF concentrations in peritoneal fluid correlated with LF, Fe and TF plasma concentrations in the group without endometriosis. Statistical analysis divided into mild and severe endometriosis demonstrated a correlation of LF with Fe and with FT in peritoneal fluid in the group with severe endometriosis with a simultaneous correlation of plasma LF with plasma Fe, regardless of the severity of endometriosis.

In Table 6, by calculating the ratios (concentration of the parameter obtained in peritoneal fluid to that obtained in plasma), the multiplicity of the difference in concentration of the determined parameters between the clinical materials was assessed. In contrast to TF, for which the ratios were the most stable, with a mean value of about 0.7, increases in the ratios above 1 for LF, FT and Fe indicate differentiation for each of these parameters (higher peritoneal fluid concentration). The highest increase in the ratio for ferritin, differentiates the group of women with and without endometriosis (*p* < 0.05).

**Figure 2:**
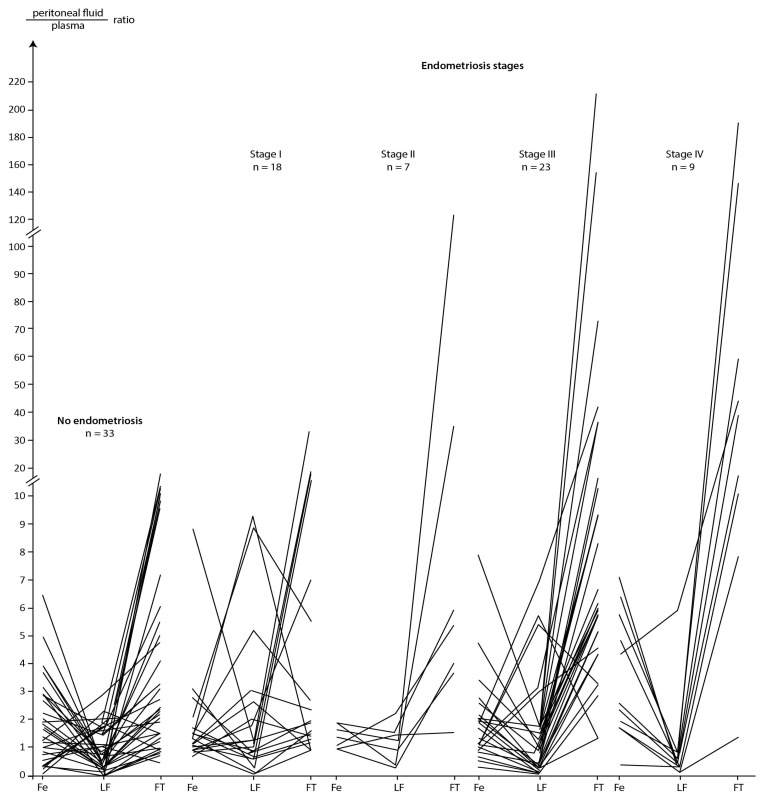
Peritoneal fluid/plasma ratios between Fe, LF, and FT in group without and subsequent stages of endometriosis.

A graphical presentation of the ratios of Fe, LF and FT concentrations between peritoneal fluid and plasma for the group without endometriosis and in subsequent stages of disease is presented in Figure 2.

Results graphically presented in Figure 2 confirms the significantly higher FT ratios in the group with endometriosis compared to that in the group without endometriosis (*p* = 0.001 in Table 6) additionally this figure shows that LF ratio values oscillated between two ranges, i.e., when LF ratio < 1.0 or when LF > 1.0. Although the ratios for LF did not differentiate between patients with and without endometriosis (*p* = 0.998 in Table 6), because in the group without and with endometriosis ratios equal to and above 1.0 represented 48% (16/33) and 46% (26/57) of the number of patients, respectively, the decreasing trend of this ratio as the severity of endometriosis progressed is notable. This is demonstrated by the fact that LF ratio values above 1.0 accounted for 61% (11/18), 57% (4/7), 43% (10/23) and 11% (1/9) of the number of patients in successive stages of endometriosis (Figure 2). In addition, statistical analysis has showed significantly higher LF ratios in stage I versus stage IV of endometriosis (*p* = 0.047). Analysing Figure 2, it can be concluded that the more advanced the degree of endometriosis, the lower the LF ratio is with a significant increase in the FT ratio. A further conclusion could be that LF ratios above 1.0 are associated with relatively lower ratios for Fe and FT. Additionally, the ratio values for LF were negatively statistically significantly correlated with those for Fe (Rs = −0.360) in the group without endometriosis (for the clarity of the presented results, an overall analysis of the correlation for the quotients is not presented in this paper).

## 3. Discussion

The presented results compare, for the first time, the concentrations of acute phase proteins, also known as iron-binding proteins (LF, TF, FT) and the concentration of iron as a panel of parameters measured simultaneously in plasma and peritoneal fluid in patients with and without endometriosis. The research model used aimed to simultaneously investigate the two different spaces in which these parameters are physiologically present.

LF is a control point in homeostasis functioning as a sensor of immunological performance related to pathology [26]. Taking this into account, we wanted to assess the contribution of this unique protein to the balance related to iron metabolism in endometriosis—a multi-causal, insidious, years-developing disease leading to chronic inflammation.

The lack of significant changes in the studied parameters in plasma suggests the impossibility of using their determinations in routinely used clinical material for diagnosing and assessing the stage of endometriosis. In contrast, the demonstration of these changes for ferritin in peritoneal fluid, a non-standard material, indicates the possibility of using this parameter in the diagnosis of the degree of the disease. Simultaneous inclusion of two clinical materials in the analysis and determination of ratios of peritoneal fluid to plasma concentration confirmed the involvement of ferritin in the successive stages of endometriosis, but in addition revealed LF as a potential “hidden player” collaborating with ferritin and Fe in the evaluation of this disease.

LF that is present in plasma and peritoneal fluid is mainly derived from neutrophils and in physiological states maintains blood concentrations at low levels of 0.2–0.6 µg/mL with a transient 100-fold increases after activation of neutrophils [30]. The plasma LF concentrations obtained in this study did not exceed 0.6 µg/mL in 24% (8/33 of the number of patients) for the groups without and 23% (13/57 of the number of patients) with endometriosis. In the case of LF determinations in peritoneal fluid, our results are partially consistent with studies [34,35] where the authors also showed no significant differences between the control group and patients with endometriosis [35]. However, they showed lower LF levels in women with benign endometriosis (stage I) compared to stages III or IV [34] and lower LF levels in patients with stage I compared to controls and to those in stage III/IV [35]. In our study, LF concentrations in the peritoneal fluid of patients with endometriosis were the highest in the first stage of the disease with a tendency to decrease in subsequent stages but without statistical significance, in contrast to ferritin and Fe, for which concentrations increased, as highlighted in other works mainly in the context of Fe [24,25].

LF as a well-established antioxidant [16,26,31]. The correlations with Fe in plasma demonstrated in this paper, for both mild and severe forms of endometriosis, and in peritoneal fluid, for severe forms of the disease, may contribute to reducing oxidative stress, particularly exacerbated in severe endometriosis [24,36]. Additionally, in an in vitro model [30] using polymorphonuclear leucocytes (PMNs) harvested from blood, Zhao et al. demonstrated that PMNs release LF after in vitro exposure to erythrocytes. In women with endometriosis, it is a natural phenomenon to periodically present a large number of erythrocytes in the peritoneal fluid as well as their degradation products including those derived from the breakdown of heme [36,37], which help to explain the lack of correlation between plasma LF and LF in peritoneal fluid in women with endometriosis and the demonstration of this correlation in women without endometriosis. Another experimental model [38] demonstrated LF’s ability to inhibit neutrophil migration, which is an additional anti-inflammatory feature of LF and is independent of its iron-chelating activity. Considering the above, it can be asked why patients in the stage IV of endometriosis (except one) had much lower concentrations of LF in peritoneal fluid compared to plasma, while this condition is characterized by a potentially large number of erythrocytes which, according to the Zhao et al. [30], become a stimulus for the release of LF from neutrophils. Another in vitro study [39] demonstrated that LF was able to inhibit neutrophil spontaneous apoptosis and showed that the anti-apoptotic effect was entirely dependent upon its iron-saturation status. In the last stage of endometriosis, we expect maximum iron saturation of LF which means that this LF has a minor effect on prolonging the life of neutrophils, which are the shortest-lived cell in the body (about 5 days) dying spontaneously by apoptosis [39]. In addition, in a study [40], the authors showed that LF is a survival factor for neutrophils in rheumatoid synovial fluid. The cited studies, conducted on experimental models, provided our work with a valuable basis for interpreting the obtained results.

The question arises of whether, from the patient’s perspective, it is more beneficial if the concentration of LF in the peritoneal fluid is at least slightly higher than that in the plasma. From our study, we conclude that if the fluid-to-plasma ratio for LF is significantly above 1.0 then the Fe and FT ratios are generally reduced and close to 1.0 or below. This needs to be confirmed on a larger group of patients and the metabolic background has to be identified. In the group without endometriosis, we additionally demonstrated a negative correlation between the ratio for LF and the ratio for Fe, which may suggest a close but concentration-dependent relationship between these two parameters. In addition, only this group of patients showed a correlation of LF concentrations between plasma and peritoneal fluid.

The demonstration in this work of ratios of peritoneal fluid to plasma concentrations represented a combination of two different environments (physiological spaces) and a deeper penetration into the body space that requires invasive sampling of clinical material. Peritoneal fluid is mainly the result of ovarian exudation and plasma transudate [34,41]. The concept of using ratios is logical and has been used previously in a paper [41] in the context of endometriosis.

In the presented study, FT concentrations were particularly “overproduced” in the peritoneal fluid. FT is an important protein for iron metabolism, which in our study was statistically significantly correlated with LF in the peritoneal fluid of patients with severe endometriosis (stages III and IV). The question is why, especially in the severe stage of the disease, the positive acute phase proteins (LF, FT) differ markedly in concentrations in the peritoneal fluid compared to the mild stage and also have a strong correlation with each other. In the context of our study, the experimental work [15] where the Balla et al. showed that endothelial cells synthesize FT to limit the reactivity of heme-derived intracellular iron becomes valuable. The high FT concentrations obtained in this work could probably be a defensive response to low LF concentrations in the severe stage of endometriosis compared to the mild stage.

Thus, in the peritoneal fluid, LF and FT control reactive free Fe and suppress oxidant damage. In addition, a high level of heme impairs the phagocytosis of peritoneal macrophages which leads to impaired elimination of endometrial stromal cells and formation of ectopic endometrial lesions [17]. Would exogenous LF be able to help restore iron balance in women with endometriosis? The answer to this question would be of great value to physicians who care for these patients.

An interesting observation from this work is also the demonstration that proteins of similar molecular weight (LF, TF) belonging to the same family [20,42] show significant discrepancies in peritoneal fluid to plasma concentration ratios. This indicates greater individuality and the influence of the environment in determining LF concentrations in the peritoneal fluid.

As highlighted by the Wong et al. [40] the ability of the iron chelators (LF, TF) to enter neutrophils could determine their ability to chelate the intracellular labile iron pool and delay neutrophil spontaneous apoptosis. From these two chelators, only LF has specific receptors on mature neutrophils and by chelating Fe delays neutrophil apoptosis, hence LF becomes the main “player” in this process. Patients in stage IV of endometriosis are particularly vulnerable to severe oxidative stress by significantly reduced LF concentrations with potentially maximum Fe saturation and with demonstrated high Fe concentrations in the peritoneal fluid, but as our studies have shown, this deregulated iron balance is trying to be repaired by FT. Additionally, in vitro studies have shown that endometrial stomal and epithelial cells are able to incorporate TF and metabolize it into FT [22], and this may be the reason why TF ratios had similar values in the group without and with endometriosis.

The strength of the work is the precisely collected material according to WERF standards. The sampling procedure, especially of the peritoneal fluid, was carried out very carefully with particular regard to the purity of the obtained samples. Weaknesses of the work include the small study group, while the promising results offer the prospect of expanding the study in the future.

## 4. Materials and Methods

### 4.1. Subjects

The study included 90 women aged between 19 and 40 years (31.0 ± 5.1 years) who qualified for diagnosis of endometriosis on the basis of clinical examination. The exclusion criteria were non-regularly menstruating patients (more than 35 or less than 25 days), age at inclusion below 18 or over 40 years old, patients on any form of hormonal therapy during the last 3 months before laparoscopy, malignant or suspected malignant disease, autoimmunological disease, previous and/or current pelvic inflammatory disease, any prior history of pelvic surgery, uterine fibroids, or polycystic ovaries.

The cycle phase was calculated based on the last menstrual period and the average length of the menstrual cycle. Moreover, the phases of the menstrual cycle in women with and without endometriosis were determined by the histological dating of the eutopic endometrial samples collected simultaneously with pathological lesions [43].

A gynaecological examination and vaginal ultrasound were performed before laparoscopy. Diagnostic laparoscopy was performed by trained gynaecologists with a detailed view of the uterus, fallopian tubes, ovaries, pouch of Douglas and pelvic peritoneum.

Patients with endometriosis were diagnosed through laparoscopic findings according to the revised American Fertility Society classification of endometriosis and each case was confirmed through histopathology. Laparoscopic examination confirmed the diagnosis of endometriosis in 57 women. According to the revised American Fertility Society classification of endometriosis [44], stage I was present in 18 cases, stage II in 7, stage III in 23, and stage IV in 9 cases. In 33 women the diagnosis of endometriosis was not confirmed. All patients underwent surgery for infertility, pelvic pain, or ovarian cysts and were asked to complete the World Endometriosis Research Foundation (WERF) clinical questionnaire. Written informed consent was obtained from all patients and the study was approved by the Ethical Committee of the Warsaw Medical University (KB/223/2017).

### 4.2. Material

Material was collected according to WERF standards in a multicenter, cross-sectional study conducted at eight departments of obstetrics and gynecology in Poland between 2018 and 2019: Department of Obstetrics and Gynecology, Medical University of Warsaw; Angelius Provita Hospital in Katowice; Department of Gynecology, Division of Infertility and Reproductive Endocrinology, Obstetrics and Gynecological Oncology at Poznan University of Medical Sciences; Department of Obstetrics and Gynecology, Central Clinical Hospital of the Ministry of Interior in Warsaw; Clinic of Obstetrics and Gynecology, Provincial Combined Hospital in Kielce; Department of Surgical Gynecology and Oncology, Medical University of Lodz; Department of Gynecology and Obstetrics, Provincial Hospital in Przemyśl; Department of Gynecology, Gynecology Oncology, and Obstetrics, Institute of Medical Sciences, Medical College of Rzeszow University.

The collected material included: *plasma*, obtained after centrifugation of blood taken from the ulnar vein into a tube with EDTA collected prior to laparoscopy and *peritoneal fluid*, obtained by careful aspiration using a Veress needle under direct visualization, taken immediately after insertion of the laparoscope to avoid blood contamination. Only clear, non-bloody contaminated fluid was collected.

Clinical materials were centrifuged for 10 min at 4° C (peritoneal fluid at 1000× *g* and plasma at 2500× *g*) then divided into smaller tubes and stored at −80° C till further use.

### 4.3. Laboratory Methods

Concentrations of LF were measured by enzyme-linked immunosorbent assay (ELISA) using a commercially available AssayMax^TM^ Human Lactoferrin ELISA Kit (Assaypro LLC–St. Charles, MO, USA, www.assaypro.com, accessed on 1 January 2020). The ELISA tests were performed in duplicate according to the manufacturer’s instructions. Lactoferrin concentrations were expressed in µg/mL.

Transferrin and ferritin concentrations were determined by immunoturbidimetric assay, in the case of FT with latex particle amplification. Iron concentration was determined by a ferrozine colorimetric assay without deproteinization. Measurements were performed on a Cobas 8000 biochemical analyzer (c7002/c502) (Roche Diagnostics, Basel, Switzerland) using branded reagents and calibrators.

### 4.4. Statistical Analysis

In order to determine the relationship of LF with Fe, TF, and FT in women with endometriosis, statistical analysis using TIBCO Statistica v. 14.0 and JAMOVI software was performed. Firstly, the normality of the data in subgroups in regard to endometriosis status (present/absent), endometriosis stage (grades I–IV), and type of fluid (plasma/peritoneal fluid) was assessed. As the distributions in many samples were highly skewed and small sample sizes in subgroups made the application of the central limit theorem impossible, alternative nonparametric tests were used. For paired samples, Wilcoxon signed rank tests were calculated, whereas for independent samples *U*-Mann Whitney and Kruskal Wallis tests were used. For assessing correlations between numerical variables Spearman’s correlation coefficient was used. For nominal variables, the χ^2^ test of independence was calculated, with Yate’s correction when necessary. The global statistical significance level was assumed as *p* < 0.05. Data are presented in Table 1, Table 2, Table 3, Table 4, Table 5 and Table 6 and in Figure 1.

## 5. Conclusions

In conclusion, our results demonstrated that LF is a physiologically important link in the pool of iron-binding proteins (FT, TF) and is in communication with free Fe. Significant increases in peritoneal fluid Fe and FT concentrations without their changes in plasma indicate the local involvement of these parameters in the regulation of iron homeostasis in patients with endometriosis. The peritoneal fluid environment is more loaded with toxic free iron than plasma. The concomitant very high FT ratios, especially in the more advanced stages of endometriosis, raise the assumption of the most active, protective involvement of this protein in this microenvironment and the loss of this role by LF. Thus, the simultaneous evaluation of plasma and peritoneal fluid concentrations of these parameters and the calculated values of peritoneal fluid-to-plasma ratios provide a more accurate picture of the disorder that is endometriosis.

## Figures and Tables

**Table 1:** Characteristics of the study (endometriosis) and control group (no endometriosis).

Variable	No Endometriosis (*n* = 33)	Endometriosis (*n* = 57)	*p*
Age, (mean ± SD)	30.52 ± 5.95	31.35 ± 4.63	0.906
Pelvic pain, *n* (%)	8 (24.24)	1 (1.75)	0.002
Primary infertility, *n* (%)	6 (18.18)	16 (28.07)	0.293
Secondary infertility, *n* (%)	6 (18.18)	2 (3.51)	0.049
Endometrial cysts, *n* (%)	-	18 (31.58)	-
Stage of endometriosis, *n* (%):			-
I	-	18 (31.58)	
II	-	7 (12.28)	
III	-	23 (40.35)	
IV	-	9 (15.79)	
Phase of cycle, *n* (%):			0.164
Follicular	25 (75.76)	35 (61.40)	
Luteal	8 (24.24)	22 (38.60)	

Note: Age data are presented as mean ± standard deviation (mean ± SD) and *p*-value denotes significance for the Mann–Whitney U test. The categorical data are presented as number (%). For nominal variables, the χ^2^ test of independence was calculated.

**Table 2:** Comparison of four variables between plasma and peritoneal fluid, regarding the presence of endometriosis.

Variables	All (*n* = 90)		*p* ^1^	No Endometriosis (*n* = 33)		*p* ^2^	Endometriosis (*n* = 57)		*p* ^3^
	Plasma	Peritoneal Fluid		Plasma	Peritoneal Fluid		Plasma	Peritoneal Fluid	
Lactoferrin (µg/mL)	1.71 ± 1.75 (0.16–8.92) 1.07	1.35 ± 1.33 (0.11–5.19) 0.92	0.220	1.59 ± 1.76 (0.37–8.92) 0.97	1.21 ± 1.15 (0.12–4.57) 0.91	0.326	1.77 ± 1.75 (0.16–6.99) 1.15	1.43 ± 1.43 (0.11–5.19) 0.98	0.397
Ferritin (µg/mL)	0.05 ± 0.04 (0.01–0.25) 0.04	0.92 ± 2.73 (0.01–18.62) 0.16	<0.001	0.04 ± 0.02 (0.01–0.10) 0.03	0.16 ± 0.21 (0.02–0.94) 0.08	<0.001	0.06 ± 0.05 (0.01–0.25) 0.05	1.36 ± 3.36 (0.01–18.62) 0.22	<0.001
Transferrin (mg/dL)	229.10 ± 44.89 (84.00–375.00) 227.00	150.80 ± 37.9 (18.00–254.00) 150.00	<0.001	229.1 ± 45.60 (84.00–307.00) 231.00	152.80 ± 38.90 (75.00–234.00) 148.00	<0.001	229.00 ± 44.80 (106.00–375.00) 227.00	149.60 ± 37.60 (18.00–254.00) 152.00	<0.001
Iron (µg/dL)	55.60 ± 40.30 (7.00–245.00) 46.50	73.70 ± 36.10 (12.00–242.00) 70.00	<0.001	53.70 ± 42.70 (9.00–215.00) 40.00	64.10 ± 32.40 (12.00–170.00) 62.00	0.089	56.70 ± 39.20 (7.00–245.00) 50.00	79.30 ± 37.30 (22.00–242.00) 72.00	<0.001

Note: *p*^1^, *p*^2^, *p*^3^:—*p*-values from Wilcoxon signed rank test for dependent observations for whole group, group without endometriosis and group with endometriosis respectively. Data are presented as mean ± SD (min–max) median. Conversion factors for iron: µg/dL × 0.179 = µmol/L, for transferrin: µg/dL × 0.125 = µmol/L.

**Table 3:** Comparison of lactoferrin, ferritin, transferrin and iron concentrations in serum and peritoneal fluid between different endometriosis stages.

Variables	Endometriosis Stage									
	Plasma					Peritoneal Fluid				
	Stage I (*n* = 18)	Stage II (*n* = 7)	Stage III (*n* = 23)	Stage IV (*n* = 9)	*p* ^1^	Stage I (*n* = 18)	Stage II (*n* = 7)	Stage III (*n* = 23)	Stage IV (*n* = 9)	*p* ^2^
Lactoferrin (µg/mL)	1.48 ± 1.60 (0.27–6.05) 0.85	1.99 ± 1.94 (0.20–5.91) 1.33	1.79 ± 1.94 (0.16–6.99) 1.09	2.17 ± 1.58 (0.26–5.28) 1.78	0.388	1.94 ± 1.82 (0.15–5.19) 0.95	1.73 ± 1.69 (0.28–4.25) 1.26	1.09 ± 1.13 (0.11–4.75) 0.64	1.03 ± 0.59 (0.12–1.54) 1.38	0.461
Ferritin (µg/mL)	0.06 ± 0.04 (0.01–0.13) 0.05 *	0.06 ± 0.04 (0.01–0.11) 0.06 *	0.06 ± 0.05 (0.01–0.25) 0.04 *	0.08 ± 0.06 (0.04–0.23) 0.06 *	0.601	0.15 ± 0.11 (0.04–0.48) 0.13 *^a,b^	0.59 ± 0.52 (0.01–1.55) 0.47 *	1.50 ± 3.83 (0.07–18.62) 0.41 *^a^	4.03 ± 5.17 (0.33–15.02) 2.39 *^b^	<0.001
Transferrin (mg/dL)	214.30 ± 39.60 (106.00–286.00) 217.00 *	251.50 ± 62.50 (200.00–375.00) 219.00 *	230.50 ± 46.90 (143.00–353.00) 227.00	237.00 ± 27.40 (191.00–276.00) 237.00	0.381	134.5 ± 23.0 (94.00–174.00) 138.00 *	160.0 ± 24.6 (124.00–196.00) 162.00 *	152.2 ± 44.6 (18.00–254.00) 155.00 *	165.00 ± 44.00 (72.00–234.00) 164.00 *	0.026
Iron (µg/dL)	45.50 ± 22.34 (7.00–81.0) 43.00 *	51.00 ± 19.94 (35.00–93.00) 46.00	64.30 ± 36.83 (11.00–155.00) 57.00	64.22 ± 71.90 (17.00–245.00) 42.00	0.473	58.11 ± 18.88 (26.00–108.00) 58.00 *^a,b^	68.43 ± 20.94 (47.00–110.00) 68.00 ^c^	82.52 ± 28.75 (22.00–146.00) 86.00 ^a^	122.00 ± 56.54 (72.00–242.00) 100.00 ^b,c^	<0.001

Note: *p*^1^, *p*^2^:—*p*-values from Kruskal-Wallis nonparametric tests for plasma and peritoneal fluid, respectively. Medians with the same letter index do differ significantly from each other, Dwass-Steel-Critchlow- Fligner (DSCF) pairwise post-hoc comparisons. *—a statistically significant difference of concentration between plasma and peritoneal fluid for specified variable and stage; Wilcoxon signed rank test for dependent observations. Data are presented as mean ± SD (min–max) median. Conversion factors for iron: µg/dL × 0.179 = µmol/L, for transferrin: µg/dL × 0.125 = µmol/L.

**Table 4:** Spearman’s rank correlations for lactoferrin (LF), ferritin (FT), iron (Fe) and transferrin (TF) for plasma and peritoneal fluid in groups with (E, *n* = 57) and without (C, *n* = 33) endometriosis only statistically significant coefficients presented.

	Variables	Group	Plasma				Peritoneal Fluid			
			LF	FT	Fe	TF	LF	FT	Fe	TF
Plasma	LF	C		0.37			0.36		0.55	0.38
		E			0.52	0.28				
	FT	C	0.37							
		E				−0.45				
	Fe	C								
		E	0.52			0.39				
	TF	C								
		E	0.28	−0.45	0.39					0.52
Peritoneal fluid	LF	C	0.36		0.55	0.38				
		E								
	FT	C								
		E							0.60	0.30
	Fe	C								
		E						0.60		0.36
	TF	C								
		E				0.52		0.30	0.36	

**Table 5:** Spearman’s rank correlations for lactoferrin (LF), ferritin (FT), iron (Fe) and transferrin (TF) for plasma and peritoneal fluid in group with endometriosis by mild (stages I and II, *n* = 25) and severe (stages III and IV, *n* = 32), only statistically significant coefficients presented.

	Variables	Stage	Plasma				Peritoneal Fluid			
			LF	FT	Fe	TF	LF	FT	Fe	TF
Plasma	LF	I and II			0.62					
		III and IV			0.49					
	FT	I and II								
		III and IV				−0.57				
	Fe	I and II	0.62						0.60	
		III and IV	0.49			0.50				
	TF	I and II								0.42
		III and IV		−0.57	0.50					0.50
Peritoneal fluid	LF	I and II								
		III and IV						0.37	0.39	
	FT	I and II							0.44	
		III and IV					0.37		0.63	
	Fe	I and II			0.60			0.44		
		III and IV					0.39	0.63		
	TF	I and II				0.42				
		III and IV				0.50				

**Table 6:** Comparison between ratios of peritoneal fluid variables’ concentrations to plasma for the whole group and in regard to the presence of endometriosis.

Variables (Peritoneal Fluid/Plasma)	Peritoneal Fluid to Plasma Ratio			*p*
	All (*n* = 90)	No Endometriosis (*n* = 33)	Endometriosis (*n* = 57)	
Lactoferrin ratio	1.41 ± 1.79 (0.04–9.24) 0.78	1.02 ± 0.73 (0.04–2.85) 0.76	1.64 ± 2.15 (0.04–9.24) 0.79	0.998
Ferritin ratio	17.82 ± 39.03 (0.45–214.02) 4.41	4.11 ± 4.14 (0.45–15.34) 2.40	25.77 ± 47.29 (0.86–214.02) 5.76	0.001
Transferrin ratio	0.68 ± 0.19 (0.09–1.55) 0.68	0.69 ± 0.24 (0.29–1.55) 0.67	0.67 ± 0.17 (0.09–1.38) 0.68	0.847
Iron ratio	1.98 ± 1.66 (0.17–8.86) 1.48	1.87 ± 1.42 (0.19–6.44) 1.43	2.05 ± 1.79 (0.17–8.86) 1.54	0.867

Note: *p—p*-values from *U*-Mann- Whitney test for independent samples between group without endometriosis and group with endometriosis. Data are presented as mean ± SD (min–max) median.

## Data Availability

The data presented in this study are available on request from the corresponding author. The data are not publicly available due to privacy.
